# Glymphatic system dysfunction in young adults hospitalized for an acute psychotic episode: a preliminary report from a pilot study

**DOI:** 10.3389/fpsyt.2025.1653144

**Published:** 2025-08-15

**Authors:** Tommaso Barlattani, Dalila De Luca, Sara Giambartolomei, Antony Bologna, Antonio Innocenzi, Federico Bruno, Valentina Socci, Maurizio Malavolta, Alessandro Rossi, Domenico de Berardis, Alessandra Splendiani, Francesca Pacitti

**Affiliations:** ^1^ Department of Biotechnological and Applied Clinical Sciences (DISCAB), University of L’Aquila, L’Aquila, Italy; ^2^ Mental Health Department, Azienda Sanitaria Locale 4 Teramo, Teramo, Italy

**Keywords:** glymphatic system, psychotic disorders, schizophrenia, diffusion tensor imaging, aquaporin-4, astrocytes, cerebrospinal fluid, extracellular fluid

## Abstract

The glymphatic system is integral to the elimination of metabolic waste from the central nervous system and has been extensively studied in relation to neurodegenerative and cerebrovascular diseases. Emerging evidence suggests that glymphatic dysfunction may also play a role in psychiatric disorders, including psychosis and schizophrenia. This pilot study investigated glymphatic clearance in young adults hospitalized for an acute psychotic episode using the diffusion tensor imaging (DTI)-derived ALPS (Along Perivascular Spaces) index. Initially, thirteen patients aged 18–30 were recruited; however, only nine completed the DTI protocol due to severe psychiatric symptoms and practical constraints. A non-psychiatric control group comprising twelve individuals was subjected to the same imaging protocol, though precise age matching was not implemented. The study’s findings indicated significant reductions in the ALPS index among patients compared to controls, supporting the hypothesis of impaired glymphatic function during acute psychotic episodes. No significant associations were found between glymphatic clearance and sleep quality, disorganized thinking, or general cognitive functioning. Despite the limitations related to the small sample size, these preliminary results highlight the necessity of further research into the glymphatic system’s involvement in psychosis. Larger-scale studies are required to elucidate the clinical implications and pathophysiological mechanisms linking glymphatic dysfunction to acute psychosis and early psychotic disorders.

## Introduction

The glymphatic system is a sleep-enhanced, astrocyte-driven paravascular network that admits cerebrospinal fluid along peri-arterial spaces, exchanges it with interstitial fluid through aquaporin-4 (AQP4) rich astroglial end-feet, and directs solute-laden efflux toward peri-venous and meningeal lymphatic pathways. (1, 2). The efficiency of glymphatic clearance depends critically on the perivascular polarization of astrocytic AQP4 water channels and rises by about 60% during delta-rich slow-wave (N3) sleep, when synchronous cortical oscillations and low-frequency vasomotion enhance cerebrospinal-to-interstitial fluid exchange (2, 3). Fragmentation or deprivation of this sleep stage blunts the characteristic cerebrospinal-fluid waves, slows solute wash-out and yields higher overnight brain β-amyloid levels (4, 5).

Glymphatic insufficiency is well documented in neurodegenerative and vascular ageing models (6, 7); however, emerging work has extended this paradigm to psychiatric disorders (8; 9). The glymphatic dysfunction stands out as a “final common pathway” through which sleep and immune dysregulation drive neuroprogression, also in psychiatric disorders (9, 10). In parallel, pro-inflammatory cytokines elevated in these illnesses depolarize AQP4 and reduce glymphatic flow, creating a mechanistic connection between the immune system and sleep dimensions (11–13).

In psychosis, a self-reinforcing loop links neurotransmitter imbalance; dopamine, serotonin, glutamate, GABA; glial-driven neuroinflammation, HPA-axis hyperactivity, gut-brain dysbiosis, oxidative stress, and mitochondrial dysfunction (14). This cascade can be initiated and intensified by impaired glymphatic clearance of neurotoxic waste (10, 15). Case-control genetic studies show that polymorphisms in AQP4, the main astrocytic water channel involved in glymphatic flux, are linked with an increased risk of schizophrenia, higher serum S100B levels, and more severe negative symptoms (16, 17). This association suggests that channel dysfunction may reduce the threshold for acute psychotic episodes (16, 17).

Diffusion-tensor imaging along the perivascular space (DTI-ALPS), an MRI metric that measures the direction of water movement within perivascular channels (18), supports this model by consistently showing lower ALPS indices in individuals with psychosis. A lower ALPS index has been observed in minimally medicated first-episode psychosis patients (19). This reduction correlates with the duration of schizophrenia illness and macromolecule accumulation in larger cohorts (20), and diminished glymphatic flow has been linked to cognitive impairment in patients with schizophrenia (21). These findings have been replicated in independent early-psychosis datasets using different imaging methods, such as BOLD CSF, to estimate glymphatic dysfunction (22).

Acute psychosis, characterized by hallucinations, delusions, and disorganized thought, frequently co-occurs with significant sleep-wake disruption and heightened inflammatory and stress-axis responses (19, 23, 24). Increasing evidence of glymphatic dysfunction in established psychotic disorders (19–22) suggests the possibility that clearance deficits may be detectable during the acute phase and could potentially act as an upstream amplifier of the aforementioned neurochemical, inflammatory, and metabolic loops.

To date, no study has investigated glymphatic function in acutely psychotic hospitalized patients. In this pilot study, we assessed DTI-ALPS, which measures anisotropic water diffusion in periventricular channels crucial for glymphatic flow, in young adults (18–30 years) experiencing their first or recurrent acute psychotic episode. Of 13 recruited patients, 9 completed high-quality DTI despite challenges in an acute psychiatric ward; 12 psychiatrically healthy volunteers served as controls. We also explored correlations between ALPS values and clinical indices of sleep quality, thought disorganization, and global cognition. Our data offer an initial look at glymphatic involvement in early-stage psychosis, while highlighting the challenges of imaging severely ill populations.

## Methods

### Participants

Between January 2024 and May 2025, thirteen patients aged 18–30 years were recruited following their admission to the Psychiatric Inpatient Unit (Servizio Psichiatrico di Diagnosi e Cura, SPDC) of San Salvatore Hospital in L’Aquila due to an acute psychotic episode attributable to a DSM-defined psychotic spectrum disorder, such as schizophreniform disorder, schizoaffective disorder, or other non-organic psychoses.

All participants were receiving antipsychotic pharmacotherapy as part of standard inpatient care at the time of recruitment and neuroimaging. All participants underwent urine toxicology screening to rule out recent substance abuse, and all tests were negative. Exclusion criteria included the presence of significant neurological conditions (e.g., acquired brain injury, demyelinating or neurodegenerative diseases), decompensated endocrine disorders, or any other medical comorbidity that could interfere with MRI evaluation. To reduce potential biases related to prolonged pharmacological exposure, individuals with a documented history of chronic psychotropic treatment, excluding their current antipsychotic therapy, were excluded.

Due to clinical instability, including severe psychomotor agitation, only nine of the thirteen initially enrolled patients were able to complete the MRI protocol. A comparison group composed of twelve individuals without any history of psychiatric disorders or major medical conditions was also enrolled, although strict age matching was not feasible due to logistical constraints and subject availability.

Psychometric assessment was conducted for all participants by trained psychiatrists with specific experience in the administration of standardized clinical tools. The Pittsburgh Sleep Quality Index (PSQI) was used to evaluate subjective sleep quality over the preceding month, with higher scores reflecting poorer sleep (25). To assess the severity of formal thought disorder, clinicians administered the Scala per la Valutazione della Disorganizzazione del Pensiero (SCADIS), a validated Italian-language instrument for quantifying disorganized thinking (26). Finally, global cognitive function was measured through the Montreal Cognitive Assessment (MoCA), which provides a comprehensive overview of executive functioning, memory, attention, language, visuospatial abilities, and orientation (28).

### Neuroimaging protocol

All brain MRIs were performed during the daytime using the same 3-Tesla scanner with a 32-channel head coil (MR750w, GE Healthcare). All MRI sessions were scheduled during the daytime hours to keep participants at the same circadian phase, because glymphatic inflow peaks during nocturnal slow-wave sleep and is markedly lower during daytime wakefulness (2, 27). This restricted window, therefore, minimized variance due to circadian fluctuations while accommodating clinical and logistical constraints, institutional availability, and ethical compliance with patient care protocols.

Individuals underwent the same brain MRI protocol, which included axial Bravo T1 FSPGR (Fast SPoiled GRadient echo; matrix 256 × 256, field of view [FOV] 25.6 cm, echo time [TE] 2.3 ms, repetition time [TR] 6.4 ms, thickness 1 mm), Axial FLAIR (Fluid Attenuated Inversion Recovery; matrix 256 × 256, FOV 25.6 cm, TE 120 ms, TR 11000ms, thickness 3 mm), DWI and DTI. DTI sequences were acquired using the following parameters: 33 diffusion directions, TR 5700 ms, TE 98 ms, parallel imaging (acceleration factor two), 3-mm slice thickness, 39 slices, matrix 128 × 128, 230 mm FOV, b value 1000 s/mm2, acquisition time 4:01 min. To obtain the ALPS index, we followed the flow chart described by Taoka et al. We processed DTI data using the software DSI Studio. An echo-planar imaging correction tool for distortion and motion correction was applied before image analysis. Spherical regions of interest (ROIs; 5 mm2) were positioned at the level of the projection fibers and the association fibers, at the level of the left lateral ventricles.

### Statistical analyses

All data analyses were carried out using R-Studio. Continuous variables were tested for normality using the Shapiro-Wilk test (p > 0.05 signifying normal distributions). Bivariate relationships among continuous measures were examined via Pearson’s correlation if both variables passed normality checks; otherwise, Spearman’s nonparametric correlation was used. We assessed the assumptions of normality (Shapiro-Wilk test) and homogeneity of variances (Levene’s test) to determine the appropriate statistical approach. Our objective was to compare the ALPS index between the two groups, adjusting for age as a covariate. Depending on compliance with these assumptions, we would have employed either an ANCOVA (Analysis of Covariance) or, alternatively, a nonparametric ANCOVA on ranks (Quade’s test). *Post-hoc* comparisons, when necessary, were performed using Bonferroni correction.

## Results

### Sample characteristics and ALPS index

Of the thirteen initially recruited patients, nine completed MRI scanning. The final patient group, all experiencing severe psychotic symptoms, had a mean ALPS index of 1.47 (SD = 0.284). The comparison group, consisting of twelve participants with no psychiatric diagnoses, had a mean ALPS index of 1.63 (SD = 0.112). Although the controls were older (mean age 34.5 ± 4.60) than the patient group, these demographic differences and the lack of strict age matching constitute a limitation. **Table 1** summarizes the sociodemographic data, whereas **Table 2** presents clinical measures for the experimental group, including PSQI (5.86 ± 1.22), SCADIS (13.91 ± 7.49), and MoCA (28.33 ± 4.27).

**Table 1:** Sociodemographic characteristics of the two samples.

Variable	Experimental group (n = 9)	Control group (n = 12)
Age (years), mean ± SD	24.4 ± 3.50	34.5 ± 4.60
Sex (F/M)	3/6 (33%/67%)	10/2 (83%/17%)
DTI-ALPS index, mean ± SD	1.47 ± 0.284	1.63 ± 0.112
Year of disease onset, mean ± SD	2020 ± 3.74	—

Continuous variables are reported as M (SD); gender is reported as f (%). Em dash (—) indicates data not available.

**Table 2:** Results of the psychometric assessment in the experimental group.

Measure	Mean ± SD	Shapiro–Wilk W	p value
PSQI score	5.86 ± 1.22	0.859	0.147
SCADIS score	13.91 ± 7.49	0.858	0.054
MoCA score	28.33 ± 4.27	0.824	0.096

PSQI, Pittsburgh Sleep Quality Index; SCADIS, Scala per la Valutazione della Disorganizzazione del Pensiero; MoCA, Montreal Cognitive Assessment.

None of the Shapiro–Wilk tests reached significance (p < 0.05), indicating approximate normality for all scales.

### Correlational analyses

**Figure 1:**
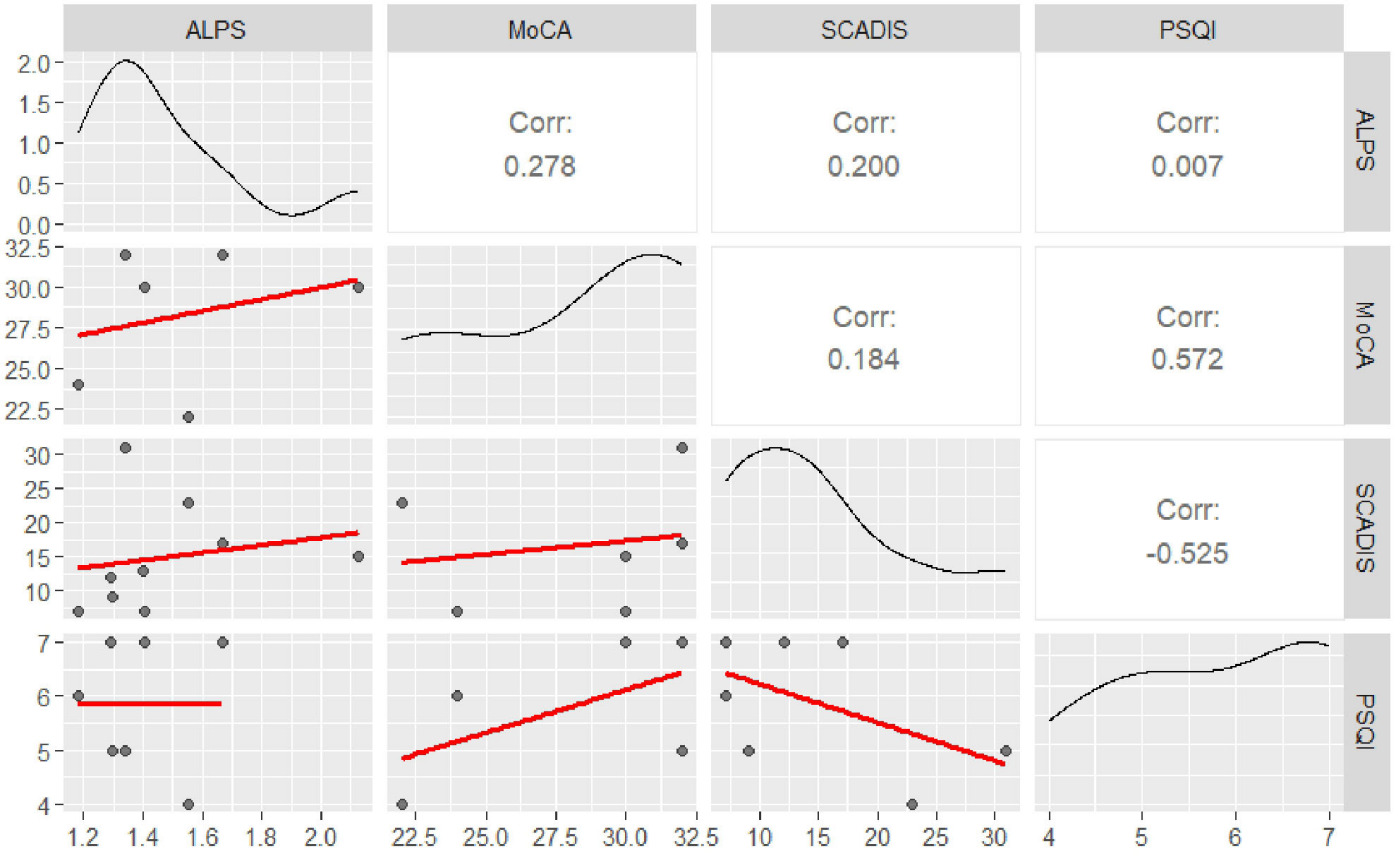
Correlation matrix of study variables. This figure displays the correlations among the analyzed variables. The upper triangle presents the strength of the correlation between each pair of variables, indicated by the correlation coefficient. The lower triangle shows scatter plots, with black dots representing individual data points and the red line indicating the linear regression (best-fit line). The main diagonal illustrates the distribution of each variable of interest (represented by a density plot).

Correlation analyses showed no significant relationships between ALPS index PSQI (r(5) = 0.007, p = 0.989), SCADIS (r(7) = 0.200, p = 0.606), and MoCA (r(4) = 0.278, p = 0.593). The results of correlations between the ALPS Index and psychometric scales are shown in **Table 3** and graphically in **Figure 1**.

**Table 3:** Correlations between ALPS index and psychometric scores.

Measure	Pearson’s r	p value
PSQI score	0.006	0.989
SCADIS score	0.200	0.606
MoCA score	0.278	0.593

PSQI, Pittsburgh Sleep Quality Index; SCADIS, Scale for the Assessment of Disorganized Thinking; MoCA, Montreal Cognitive Assessment. p <.05.

### Comparison between patients and controls

**Figure 2:**
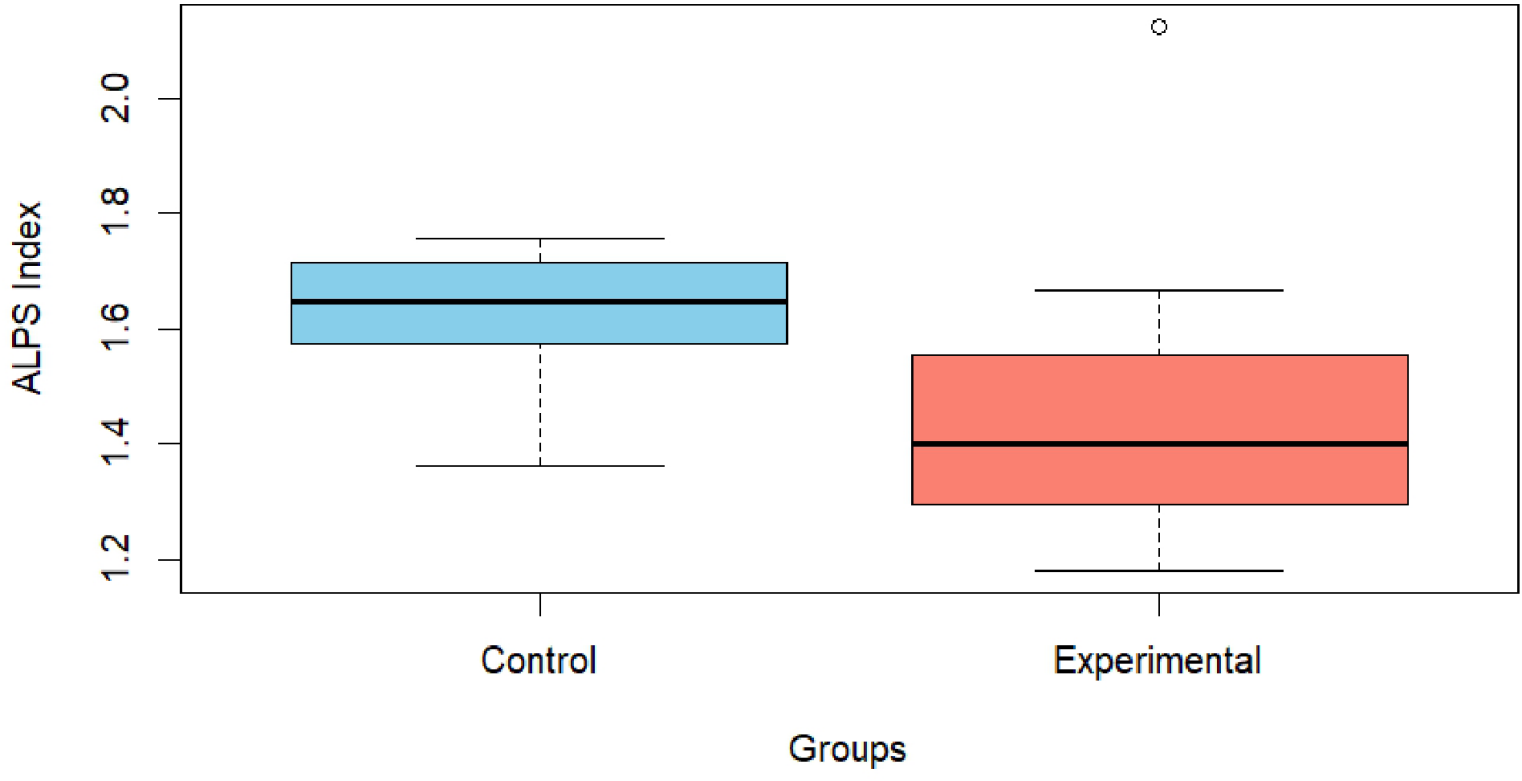
Graphical representation of the ALPS index between the control and experimental groups. The graph illustrates the observed difference between the Control group (skyblue), with a value of 1.63 ± 0.112, and the Experimental group (salmon), which presents a value of 1.47 ± 0.284.

Prior to conducting the ANCOVA, assumptions of normality (Shapiro-Wilk test: W=0.890, p=0.022) and homogeneity of variances (Levene’s test: F(1,19)=4.71, p=0.043) were assessed. Both assumptions were violated. Consequently, a nonparametric ANCOVA was employed (Quade’s test). Results from the nonparametric ANCOVA revealed a significant difference between the two groups (F(1,199)=6.59, p=0.02). This finding was further supported by *post-hoc* analyses with Bonferroni correction (mean rank difference = 5.38, p=0.047). Specifically, the experimental group had a significantly lower rank than the control group. This corresponded to mean ALPS scores of 1.474 for the experimental group and 1.629 for the control group. Further details are presented in **Table 4** and illustrated in **Figure 2**.

**Table 4:** Nonparametric ANCOVA results.

	*Estimate*	*df*	*sumsq*	*meansq*	*statistic*	*p.value*
a) Omnibus effects						
Group		1	199.111	199.111	6.587	0.020
rank_eta		1	17.760	17.760	0.587	0.454
Group:rank_eta		1	39.264	39.264	1.299	0.270
Residuals		17	513.864	30.227		
b) *Post-Hoc*						
Control - Experimental	5.380	17			2.134	0.0477

df, degree of freedom; sumsq, sum of square; meansq, means square; * *p*<0.05.

## Discussion

This pilot study presents initial evidence indicating a reduced ALPS index, suggesting potential glymphatic impairment, in young adults hospitalized for acute psychosis compared to controls. The hypothesis that the brain’s glymphatic waste-clearance system may be compromised during acute psychotic episodes is consistent with the expanding body of literature that links glymphatic dysfunction to psychiatric disorders and psychosis (8, 9, 20–22).

Abdolizadeh et al. (20) evidenced that the ALPS index continuously declines in cases of chronic schizophrenia. Likewise, Tu et al. (21) also reported decreased ALPS indices in both the left and right hemispheres and the entire brain in comparison with healthy controls and related lower ALPS values to worse global cognition. However, both Tu et al. and Adbolizadeh et al. concentrated on patients with schizophrenia who were chronically ill and undergoing antipsychotic treatment. Korann et al. (19) reported bilateral ALPS-index reductions in minimally medicated, first-episode psychosis-spectrum patients. Their findings indicate that glymphatic dysfunction is already present at illness onset, rather than arising as a consequence of antipsychotic exposure.

This evidence strengthens our interpretation and mitigates concerns that the lower ALPS values observed in our cohort could merely reflect the effects of acute antipsychotic treatment. Collectively, these convergent findings, along with our preliminary report expanding the data on the acute psychotic phase, indicate that a lower ALPS index could potentially serve as an indicative marker of psychosis across the entire spectrum of the disease.

Hua et al. showed that individuals in early psychosis exhibit both attenuated and delayed coupling between cortical BOLD fluctuations and ventricular cerebrospinal fluid (CSF) inflow, an fMRI marker of reduced glymphatic clearance, when compared with healthy controls. Weaker global BOLD–CSF coupling was further associated with poorer cognitive performance and greater symptom severity, underscoring the clinical relevance of glymphatic dysfunction. Notably, their cohort’s mean age (~24 years) reflects that of our acutely psychotic sample (24.4 years). The convergence of Hua et al.’s findings with our preliminary observation of lower ALPS indices therefore strengthens the hypothesis that glymphatic impairment is already present at illness onset in young patients.

From a pathophysiological standpoint, there is increasing evidence suggesting that glymphatic dysfunction may play a mechanistically significant role in the onset and progression of psychotic disorders, particularly during acute episodes. In the context of acute psychosis, this system appears to be functionally compromised, with accumulating data indicating that astrocyte distress plays a central role in this dysfunction. First-episode psychosis is marked by a consistent increase in serum and CSF concentrations of S100B, a calcium-binding protein that is released into the extracellular space following astrocytic membrane disruption (29). Importantly, this elevation occurs without concurrent increases in traditional markers of neuronal or myelin injury, such as GFAP, NSE, or MBP, suggesting a selective vulnerability of astrocytic compartments rather than widespread neurodegeneration (29).

Structural imaging and post-mortem studies support this model: astrocytic end-feet often appear swollen, perivascular AQP4 polarization is frequently lost, and perisynaptic processes are diminished or disorganized (30, 31). These alterations are likely to impede the laminar flow of interstitial fluid, consequently disrupting glymphatic clearance. Genetic and translational research indicates that AQP4 dysregulation plays a role in schizophrenia. Functional variants of the AQP4 gene have been linked to an increased risk of psychosis and more severe symptoms (32).

Preclinical data indicate that simply increasing AQP4 protein does not enhance glymphatic flow. On the contrary, AQP4 overexpression that lacks correct perivascular polarization reduces solute clearance and aggravates schizophrenia-like behaviors in rodents. As recent evidence clarifies, AQP4 mislocalization contributes to impaired glymphatic function rather than fully accounting for it. Rodent studies show that genetic deletion or depolarization of AQP4 reduces solute clearance by over 40% (1), and human post-mortem analyses have linked loss of perivascular AQP4 polarization to cognitive decline and amyloid accumulation (33).

More recently, Simon et al. (34) have further emphasized that AQP4-dependent solute transport critically depends on its spatial organization at the astrocyte endfeet rather than total protein abundance. Targeted genetic or pharmacological downregulation of this malpolarized AQP4 pool partly restores glymphatic flux and ameliorates those behaviors (35). Hence, our finding of lower ALPS indices in acutely psychotic patients is not the opposite of the animal evidence but its clinical counterpart: both sets of data converge on the notion that glymphatic inefficiency arises from dysfunctional or mislocalized AQP4 channels rather than from a quantitative shortfall of the protein.

These findings suggest that early astrocyte dysfunction may impair perivascular fluid exchange, leading to the accumulation of neurotoxic byproducts, increased oxidative stress, and abnormal neuroimmune responses. This glymphatic compromise directly impacts cognitive functioning. Acute psychosis often involves significant cognitive impairments in working memory, executive control, and attention (36). These issues are linked to sleep disturbances and astrocytic dysfunction. Sleep, especially slow-wave activity, drives glymphatic flow. Disrupted sleep, common in high-risk psychosis patients or during episodes, may worsen glymphatic inefficiency, creating a cycle of sleep deprivation, impaired clearance, and cognitive decline (37). The link between astrocytic integrity, glymphatic dynamics, and neurocognitive health indicates that sleep loss and astroglial pathology may exacerbate cognitive deficits in psychotic illness.

The findings of the current study, which show an altered ALPS index distribution in young adults hospitalized for acute psychosis, align with a broader model suggesting that astrocyte-driven impairment of glymphatic function may play a role in the pathophysiology of psychosis. Glymphatic failure might occur early in the disease process, potentially even before the full clinical manifestation, rather than being a downstream consequence or residual scar of chronic illness.

This pilot study offers preliminary evidence of glymphatic disruption in young adults hospitalized for acute psychosis. This finding complements the emerging body of literature that has started to associate glymphatic impairment with psychiatric disorders (9, 20–22). Despite the difficulty of imaging acutely ill patients, evidenced by only nine of thirteen completing the protocol, the results suggest a substantive alteration in ALPS index distribution relative to controls. Although our study did not show strong ties with subjective sleep quality, thought disorganization, or overall cognition, such negative results may be attributed to limited statistical power, cross-sectional design, or the specificity of the measures.

This pilot study must be interpreted in light of several limitations. First, the analytical sample was very small (n = 9) and 31 % of initially eligible patients could not tolerate MRI because of agitation, reducing power and external validity. To curb attrition, future work could employ: shorter diffusion sequences, evening scans preceded by symptom-targeted sedation, and pre-scan “mock-scanner” acclimatization sessions. The controls (34.5 ± 4.6 y) were almost 10 years older than patients (24.4 ± 3.5 y). Because ALPS normally declines with age, this imbalance works against the group difference we observed, but nevertheless increases variance. We therefore applied a non-parametric ANCOVA on age-adjusted ranks (Quade’s test), which confirmed a significant group effect (F = 6.59, p = 0.020).

This paradoxical result diverges from the well-established age-related decline in ALPS values, attributed to reduced glymphatic efficiency with advancing age (38). One plausible explanation is that pathophysiological mechanisms intrinsic to psychosis accelerate brain-aging processes from the earliest stages of the illness (39).

Large-scale neuroimaging studies consistently report an accelerated brain-age phenotype in individuals with psychotic disorders. A meta-analysis from the ENIGMA consortium found a mean brain-predicted-age difference (brain-PAD) of +3.55 years in 5,401 patients with schizophrenia (39). Similarly, a PRISMA-guided meta-analysis of 18 datasets reported a comparable brain-PAD of +3.08 years in schizophrenia and first-episode psychosis (40). A mega-analysis of 45,615 participants confirmed this effect across major psychiatric conditions, including psychotic disorders, suggesting a transdiagnostic brain ageing signature (41).

Notably, a seminal pattern recognition study estimated a brain age gap of +5.5 years already at the first episode in schizophrenia, and +1.7 years in at-risk mental states (42). Taken together, these converging findings support our interpretation that lower ALPS index values may reflect an early-onset senescent trajectory in acute psychosis.

Moreover, the study was insufficiently powered to include multiple covariates, such as gender, although clinically relevant, that could not be added without over-fitting. We prioritized age over gender because its empirical influence on ALPS is well established (38), yet acknowledge that omitting gender is a residual source of bias. Future studies need to use strict age- and sex-matching or propensity-score weighting to balance both covariates simultaneously.

Finally, the cross-sectional design precludes conclusions about causality or temporal dynamics of glymphatic impairment; longitudinal imaging with sleep and inflammatory markers will be required to clarify the mechanism.

Collectively, these observations support the notion that acute psychosis may impair glymphatic function independently of chronological age. Additionally, objective sleep metrics, such as polysomnography and inflammatory markers, were not collected, limiting the ability to dissect the mechanistic underpinnings of glymphatic alterations. Moreover, we did not compare or correlate ALPS values with smoking status, exposure to other environmental agents, hypertension, or additional potential confounders.

The intrinsic technical limitations of DTI‐based ALPS quantification warrant caution and highlight the need for longitudinal validation using complementary imaging modalities. Moreover, glymphatic activity varies across the sleep–wake cycle, restricting all scans to daytime likely underestimates the nocturnal peak in CSF–ISF exchange, and this circadian limitation should be considered when interpreting the magnitude of ALPS differences (43). Although susceptibility‐weighted imaging was not employed to localize medullary veins, previous work by Taoka and colleagues (2024) (43) indicates that the ALPS method remains reproducible even without SWI guidance.

Nonetheless, these preliminary data emphasize the necessity of further in-depth research with larger cohorts, better age-matching, longitudinal tracking, and comprehensive assessments of sleep and inflammation.

## Conclusion

Despite the inherent methodological difficulties in acquiring neuroimaging data from acutely symptomatic individuals, the present pilot study provides preliminary evidence that young adults hospitalized for acute psychosis exhibit alterations in glymphatic function indicative of impaired interstitial waste clearance. Specifically, reduced ALPS index values in patients relative to non-psychiatric controls align with a growing body of literature implicating astrocytic dysfunction, AQP4 misregulation, and elevated peripheral S100B levels in the acute phase of psychosis.

These findings suggest that glymphatic impairment may be an early and functionally relevant component of psychosis pathophysiology, potentially contributing to the accumulation of neurotoxic metabolites and the emergence of cognitive deficits. Future longitudinal research integrating sleep assessment, inflammatory markers, and targeted glymphatic interventions is warranted to determine whether restoring perivascular clearance can modulate the clinical and cognitive trajectory of psychotic illness.

## Data Availability

The raw data supporting the conclusions of this article will be made available by the authors, without undue reservation.

## References

[1] Iliff JJ Wang M Liao Y Plogg BA Peng W Gundersen GA. A paravascular pathway facilitates CSF flow through the brain parenchyma and the clearance of interstitial solutes, including amyloid β. Sci Trans Med. (2012) 4:147ra111–147ra111. doi: 10.1126/scitranslmed.3003748, PMID: 22896675 PMC3551275

[2] Xie L Kang H Xu Q Chen MJ Liao Y Thiyagarajan M. Sleep drives metabolite clearance from the adult brain. Sci (New York N.Y.). (2013) 342:373–7. doi: 10.1126/science.1241224, PMID: 24136970 PMC3880190

[3] Nedergaard M Goldman SA. Glymphatic failure as a final common pathway to dementia. Science. (2020) 370:50–6. doi: 10.1126/science.abb8739, PMID: 33004510 PMC8186542

[4] Fultz NE Bonmassar G Setsompop K Stickgold RA Rosen BR Polimeni JR. Coupled electrophysiological, hemodynamic, and cerebrospinal fluid oscillations in human sleep. Science. (2019) 366:628–31. doi: 10.1126/science.aax5440, PMID: 31672896 PMC7309589

[5] Lucey BP Holtzman DM. How amyloid, sleep and memory connect. Nat Neurosci. (2015) 18:933–4. doi: 10.1038/nn.4048, PMID: 26108720 PMC4770804

[6] Kress BT Iliff JJ Xia M Wang M Wei HS Zeppenfeld D Impairment of paravascular clearance pathways in the aging brain. Ann Neurol. (2014) 76(6):845–61. doi: 10.1002/ana.24271, PMID: 25204284 PMC4245362

[7] Lundgaard I Lu ML Yang E Peng W Mestre H Hitomi E Glymphatic clearance controls state dependent changes in brain lactate concentration. J Cereb Blood Flow Metab. (2017) 37(6):2112–2124. doi: 10.1177/0271678X16661202, PMID: 27481936 PMC5464705

[8] Barlattani T Grandinetti P Cintio AD Montemagno A Testa R D'Amelio C Glymphatic System and Psychiatric Disorders: A Rapid Comprehensive Scoping Review. Curr Neuropharmacol. (2024) 22(12):2016–2033. doi: 10.2174/1570159X22666240130091235, PMID: 39234773 PMC11333792

[9] Gao T Wang Z Tabarak S Yin Q Lu L. The glymphatic system in psychiatric disorders: a new perspective. Sci Bull. (2025) 70, S2095–9273. doi: 10.1016/j.scib.2025.05.015, PMID: 40379518

[10] Szlufik S Kopeć K Szleszkowski S Koziorowski D. Glymphatic system pathology and neuroinflammation as two risk factors of neurodegeneration. Cells. (2024) 13:286. doi: 10.3390/cells13030286, PMID: 38334678 PMC10855155

[11] Dantzer R O’Connor JC Freund GG Johnson RW Kelley KW. From inflammation to sickness and depression: when the immune system subjugates the brain. Nat Rev Neurosci. (2008) 9:46–56. doi: 10.1038/nrn2297, PMID: 18073775 PMC2919277

[12] Bauer ME Teixeira AL. Inflammation in psychiatric disorders: what comes first? Ann N Y Acad Sci. (2019) 1437:57–67. doi: 10.1111/nyas.13712, PMID: 29752710

[13] Ferini-Strambi L Salsone M. Glymphatic” Neurodegeneration: is sleep the missing key? Clin Trans Neurosci. (2024) 8 :23. doi: 10.3390/ctn8020023

[14] Rawani NS Chan AW Dursun SM Baker GB. The underlying neurobiological mechanisms of psychosis: focus on neurotransmission dysregulation, neuroinflammation, oxidative stress, and mitochondrial dysfunction. Antioxidants. (2024) 13:709. doi: 10.3390/antiox13060709, PMID: 38929148 PMC11200831

[15] Kopeć K Szleszkowski S Koziorowski D Szlufik S. Glymphatic system and mitochondrial dysfunction as two crucial players in pathophysiology of neurodegenerative disorders. Int J Mol Sci. (2023) 24:10366. doi: 10.3390/ijms241210366, PMID: 37373513 PMC10299586

[16] Wu Y-F Sytwu H-K Lung F-W. Human aquaporin 4 gene polymorphisms and haplotypes are associated with serum S100B level and negative symptoms of schizophrenia in a Southern Chinese Han population. Front Psychiatry. (2018) 9:657. doi: 10.3389/fpsyt.2018.00657, PMID: 30618856 PMC6297372

[17] Wu Y-F Sytwu H-K Lung F-W. Polymorphisms in the human aquaporin 4 gene are associated with schizophrenia in the Southern Chinese Han population: a case–control study. Front Psychiatry. (2020) 11:596. doi: 10.3389/fpsyt.2020.00596, PMID: 32676041 PMC7333661

[18] Taoka T Ito R Nakamichi R Nakane T Kawai H Naganawa S Diffusion Tensor Image Analysis ALong the Perivascular Space (DTI ALPS): Revisiting the Meaning and Significance of the Method. Magn Reson Med Sci. (2024) 23(3):268–290. doi: 10.2463/mrms.rev.20230175, PMID: 38569866 PMC11234944

[19] Korann V Panganiban KJ Stogios N Remington G Graff-Guerrero A. The dysregulation of the glymphatic system in patients with psychosis spectrum disorders minimally exposed to antipsychotics. Can J Psychiatry. (2025) 70:260–70. doi: 10.1177/07067437241290193, PMID: 39428987 PMC11562879

[20] Abdolizadeh A Torres-Carmona E Kambari Y Amaev A Song J. Evaluation of the glymphatic system in schizophrenia spectrum disorder using proton magnetic resonance spectroscopy measurement of brain macromolecule and diffusion tensor image analysis along the perivascular space index. Schizophr Bull. (2024) 50:1396–410. doi: 10.1093/schbul/sbae060, PMID: 38748498 PMC11548937

[21] Tu Y Fang Y Li G Xiong F Gao F. Glymphatic system dysfunction underlying schizophrenia is associated with cognitive impairment. Schizophr Bull. (2024) 50:1223–31. doi: 10.1093/schbul/sbae039, PMID: 38581275 PMC11349007

[22] Hua L Zeng X Zhang K Zhao Z Yuan Z. Reduced glymphatic clearance in early psychosis. Mol Psychiatry(. (2025). doi: 10.1038/s41380-025-03058-1, PMID: 40389626

[23] van Os J Kapur S. Schizophrenia. Lancet. (2009) 374:635–45. doi: 10.1016/S0140-6736(09)60995-8, PMID: 19700006

[24] Walker E Mittal V Tessner K. Stress and the hypothalamic pituitary adrenal axis in the developmental course of schizophrenia. Annu Rev Clin Psychol. (2008) 4:189–216. doi: 10.1146/annurev.clinpsy.4.022007.141248, PMID: 18370616

[25] Curcio G Tempesta D Scarlata S Marzano C Moroni F Rossini PM. Validity of the italian version of the pittsburgh sleep quality index (PSQI). Neurol Sci. (2013) 34:511–9. doi: 10.1007/s10072-012-1085-y, PMID: 22526760

[26] Brugnoli R Pacitti F Tarsitani L Troisi A Rossi A Pancheri P. Dimensione disorganizzazione in corso di schizofrenia e funzionamento sociale. Giorn Ital Psicopat. (2006) 12:208–16.

[27] Hablitz LM Plá V Giannetto M Vinitsky HS Stæger FF Metcalfe T Circadian control of brain glymphatic and lymphatic fluid flow. Nat Commun. (2020) 11(1):4411. doi: 10.1038/s41467-020-18115-2, PMID: 32879313 PMC7468152

[28] Pirrotta F Timpano F Bonanno L Nunnari D Marino S Bramanti P. Italian validation of Montreal cognitive assessment. (2015). (Milan, Italy: Springer)

[29] Steiner J Bielau H Bernstein HG Bogerts B Wunderlich MT. Increased cerebrospinal fluid and serum levels of S100B in first-onset schizophrenia are not related to a degenerative release of glial fibrillar acidic protein, myelin basic protein and neurone-specific enolase from glia or neurones. J Neurology Neurosurg Psychiatry. (2006) 77:1284–7. doi: 10.1136/jnnp.2006.093427, PMID: 17043297 PMC2077376

[30] de Oliveira Figueiredo EC Cali C Petrelli F Bezzi P. Emerging evidence for astrocyte dysfunction in schizophrenia. Glia. (2022) 70:1585–604. doi: 10.1002/glia.24221, PMID: 35634946 PMC9544982

[31] Vellucci L Mazza B Barone A Nasti A De Simone G Iasevoli F. The role of astrocytes in the molecular pathophysiology of schizophrenia: between neurodevelopment and neurodegeneration. Biomolecules. (2025) 15:615. doi: 10.3390/biom15050615, PMID: 40427508 PMC12109222

[32] Muratake T Fukui N Kaneko N Amagane H Someya T. Linkage disequilibrium in aquaporin 4 gene and association study with schizophrenia. Psychiatry Clin Neurosci. (2005) 59:595–8. doi: 10.1111/j.1440-1819.2005.01420.x, PMID: 16194264

[33] Zeppenfeld DM Simon M Haswell JD D’Abreo D Murchison C Quinn JF. Association of perivascular localization of aquaporin-4 with cognition and alzheimer disease in aging brains. JAMA Neurol. (2017) 74:91–9. doi: 10.1001/jamaneurol.2016.4370, PMID: 27893874

[34] Simon M Wang MX Ismail O Braun M Schindler AG Reemmer J. Loss of perivascular aquaporin-4 localization impairs glymphatic exchange and promotes amyloid β plaque formation in mice. Alzheimers Res Ther. (2022) 14:59. doi: 10.1186/s13195-022-00999-5, PMID: 35473943 PMC9040291

[35] Nie FY Jin RY Wu SS Yuan W Wu YW Xue SM. AQP4 is upregulated in schizophrenia and its inhibition attenuates MK-801-induced schizophrenia-like behaviors in mice. Behav Brain Res. (2024) 475:115220. doi: 10.1016/j.bbr.2024.115220, PMID: 39214422

[36] McCutcheon RA Keefe RSE McGuire PK. Cognitive impairment in schizophrenia: aetiology, pathophysiology, and treatment. Mol Psychiatry. (2023) 28:1902–18. doi: 10.1038/s41380-023-01949-9, PMID: 36690793 PMC10575791

[37] Kim J Prasad S Roshan NS Hasan BF Gill G Gunturu S. Sleep disruptions and the pathway to psychosis: An in-depth case and literature review. Clin Case Rep. (2024) 12:e9108. doi: 10.1002/ccr3.9108, PMID: 38887308 PMC11180692

[38] Hsiao WC Chang HI Hsu SW Lee CC Huang SH Cheng CH. Association of cognition and brain reserve in aging and glymphatic function using diffusion tensor image-along the perivascular space (DTI-ALPS). Neuroscience. (2023) 524:11–20. doi: 10.1016/j.neuroscience.2023.04.004, PMID: 37030632

[39] Costantidines C Han LKM Alloza C. Brain ageing in schizophrenia: evidence from 26 international cohorts via the ENIGMA Schizophrenia consortium. Mol Psychiatry. (2023) 28:1201–9. doi: 10.1038/s41380-022-01897-, PMID: 36494461 PMC10005935

[40] Ballester PL Romano MT de Azevedo Cardoso T Hassel S Strother SC Kennedy SH. Brain age in mood and psychotic disorders: a systematic review and meta-analysis. Acta Psychiatr Scand. (2022) 145:42–55. doi: 10.1111/acps.13371, PMID: 34510423

[41] Kaufmann T van der Meer D Doan NT Schwarz E Lund MJ Agartz I. Common brain disorders are associated with heritable patterns of apparent aging of the brain. Nat Neurosci. (2019) 22:1617–23. doi: 10.1038/s41593-019-0471-7, PMID: 31551603 PMC6823048

[42] Koutsouleris N Davatzikos C Borgwardt S Gaser C Bottlender R Frodl T. Accelerated brain aging in schizophrenia and beyond: a neuroanatomical marker of psychiatric disorders. Schizophr Bull. (2014) 40:1140–53. doi: 10.1093/schbul/sbt142, PMID: 24126515 PMC4133663

[43] Taoka T Naganawa S. Neurofluid dynamics and the glymphatic system: A neuroimaging perspective. Korean J Radiol. (2020) 21:1199–209. doi: 10.3348/kjr.2020.0042, PMID: 32783417 PMC7462760

